# A Novel Use of Early Radiation Therapy in the Treatment of Hyperbilirubinemia in a Patient with Primary Hepatic Lymphoma and Chronic Hepatitis C

**DOI:** 10.1155/2014/724256

**Published:** 2014-04-29

**Authors:** Venkata S. Tammana, Rehana Begum, Patricia Oneal, Hemamalini Karpurapu, Amruta Muley, Sri Lakshmi Hyndavi Yeruva, Jacquelyn Dunmore-Griffith, Eyasu Mekonen, Nabhani Hasan

**Affiliations:** ^1^Department of Internal Medicine, Howard University Hospital, 2041 Georgia Avenue, NW, Washington, DC 20060, USA; ^2^Department of Radiation Oncology, Howard University Hospital, Washington, DC, USA; ^3^Department of Radiology, Howard University Hospital, Washington, DC, USA

## Abstract

Lymphomas arising in the liver are extremely rare. Here, we describe a case of Hepatitis C virus infection with primary hepatic lymphoma (PHL) presenting with hyperbilirubinemia. A 45-year-old African American male presented with abdominal pain, pruritus, and itching for two days. CT of abdomen and pelvis with contrast showed numerous masses in the liver. The liver biopsy was consistent with diffuse large B cell lymphoma (DLBCL). Conventional chemotherapy was avoided initially because of hyperbilirubinemia. Hence, radiation therapy was given initially to reduce his bilirubin levels and tumor size. The patient was able to complete six cycles of rituximab combined with cyclophosphamide, adriamycin, vincristine, and prednisone (R-CHOP) chemotherapy and achieved a complete response verified by positron emission tomography-computed tomography (PET-CT). PHL should be considered when there are numerous space occupying liver lesions seen on imaging. Hyperbilirubinemia may be a reason for delay in treatment for some of these patients. Hence, the role of radiation therapy prior to treatment with R-CHOP is an alternative to management for stage IV diffuse large B cell lymphoma.

## 1. Introduction

Lymphomas arising in liver are extremely rare. They can be primary (extranodal lymphoma arising solely in the liver without involvement of any other organ) or secondary (secondary involvement of liver in systemic non-Hodgkin's lymphoma (NHL)). Primary hepatic lymphoma (PHL) comprises only about 0.016% of all cases of non-Hodgkin's lymphoma [1]. PHL have been described in patients with Hepatitis C virus (HCV) infection [2]. Because of nonspecific clinical symptoms or imaging pattern, PHL is often misdiagnosed as hepatocellular carcinoma, metastatic tumor, or liver abscess. Here, we present a case of PHL in a patient with HCV infection presenting with significant hyperbilirubinemia and highlight his response to radiation therapy and systemic chemotherapy.

## 2. Case Presentation

A 45-year-old African American male with a past medical history of hypertension, HCV infection, and polysubstance abuse was admitted with epigastric and right upper quadrant abdominal pain, pruritus, and itching for two days. Symptoms are associated with nonbilious vomiting, fatigue, and a 9 kg unintentional weight loss over three months. He denied any history of Hepatitis B in the past. Ten months before, he had a similar complaint where evaluation at an outside facility showed a hepatic mass. A liver biopsy at that time showed acute inflammation suggestive of an abscess with extensive chronic inflammation and fibrosis adjacent to the necrosis, moderate to severe acute and chronic periportal inflammation with portal lymphoid aggregates. Cultures from the biopsy were negative for any organisms but the patient was treated with an empiric course of vancomycin and piperacillin/tazobactam for four weeks. His social history was significant for cigarette smoking, alcohol, and intravenous illicit drug use of heroin and cocaine. The family history was significant for a brother with gastric lymphoma, now in remission. Physical exam was significant for skin excoriations, scleral icterus without any lymphadenopathy, hepatomegaly, and tenderness along the epigastrium and right upper quadrant areas.

**Figure 1:**
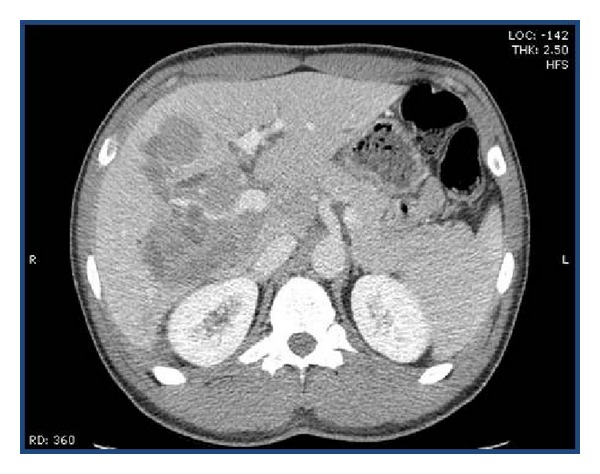
CT scan with intravenous contrast-portal venous phase showing multiple lesions in the liver.

**Figure 2:**
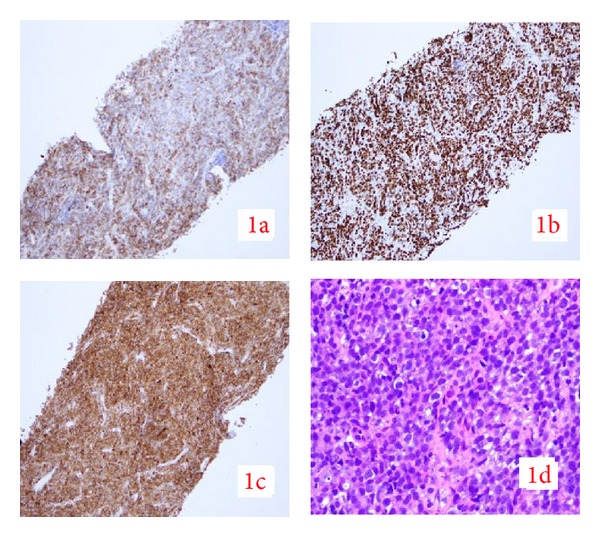
Liver Biopsy shows (1a) CD 20 positive on IHC. (1c) CD 10 positive on IHC. (1b) Ki 67 of 100% (1d) DLBCL.

Initial laboratory values showed a hemoglobin of 13.0 gm/dL, hematocrit of 37.7%, white blood cell count of 4.4 × 10^3^, and platelet count of 240 × 10^3^. His metabolic panel showed normal renal function tests with a calcium of 9.4 mg/dL. His comprehensive metabolic panel revealed a total bilirubin of 6.1 mg/dL, direct bilirubin of 4.1 mg/dL, aspartate aminotransferase (AST) of 150 U/L, alanine aminotransferase (ALT) of 142 U/L, alkaline phosphatase (ALP) of 282 IU/L, gamma-glutamyl transferase (GGT) of 385 IU/L, total protein of 7.4 g/dL, and albumin of 3.6 g/dL. His prothrombin time was 13.7 seconds with an International Normal Ratio (INR) of 1.04. Amylase and lipase levels were within normal limits. Hepatitis B panel showed immunity to Hepatitis B. His HCV RNA viral load was 422131 IU/mL (5.63 log). HCV genotype is 1b. HIV by ELISA was negative. His alpha-fetoprotein levels (AFP) were 2.2 ng/mL. Blood cultures were negative. Triphasic computed tomography (CT) of the abdomen and pelvis with contrast showed multiple masses in the liver (largest being 8.8 cm) without any cholelithiasis or extrahepatic bile duct dilatation (Figure 1). There was no significant enhancement of the hepatic lesions on arterial phase, while on portal venous phase the lesions showed slight enhancement. The spleen, pancreas, gallbladder, kidneys, and adrenals appeared within normal limits. Over his hospital course, his bilirubin levels increased to 23.3 mg/dL on eleventh day along with aspartate transaminase (AST) of 277 U/L, alanine aminotransferase (ALT) of 197 U/L, and alkaline phosphatase (ALP) of 199 IU/L (Table 1). In spite of high bilirubin levels, the patient maintained normal coagulation parameters and showed no evidence of hepatic encephalopathy. CT chest with contrast was within normal limits. Meanwhile, both upper endoscopy and colonoscopy were negative for occult malignancy. The biopsy of the liver mass done under fluoroscopic guidance was consistent with diffuse large B cell lymphoma (DLBCL). By immunohistochemistry, the cells were positive for CD 20, CD 10, and BCL-6. The Ki-67 proliferative index was 100% (Figure 2). FISH for c-myc rearrangement was negative. Bone marrow biopsy was negative for lymphoma.

With stage IVB DLBCL and elevated bilirubin levels, there was a concern for giving full dose chemotherapy, since the liver metabolizes most of the chemotherapy agents. A decision was made to do local irradiation to the liver and reevaluate his bilirubin level to determine if chemotherapy could be administered. The patient's liver received a total dose of 6 Gy in 4 fractions of 1.5 Gy each using a 2-field 3D conformal technique with 10 MV photon beam therapy on days 1, 2, 9, and 12. Initial staging positron emission tomography- (PET-) CT showed decreased metabolic activity compared with normal adjacent liver parenchyma, suggesting a positive response to treatment and no extranodal disease. Meanwhile, total bilirubin levels fell to 3.4 mg/dL (Table 2) and allowed the first dose of R-CHOP (rituximab combined with cyclophosphamide, adriamycin, vincristine, and prednisone) chemotherapy to be administered at a 50% dose reduction. The patient subsequently received six cycles of R-CHOP. After completion, beside neutropenia, no other significant adverse effects were noted. Restaging PET-CT showed no hypermetabolic activity in the liver.

## 3. Discussion

Caccamo et al. [9] defined PHL as a lymphoma localized and limited to the liver without extrahepatic involvement. The vast majority (67%) of PHL patients are middle aged men (median age 53 years) [10]. The male to female ratio is 2.3 : 1.0. Page et al. [2] reviewed twenty-four patients with PHL in which seventeen patients (71%) had right upper quadrant pain, nine patients (38%) had B symptoms (fever, night sweats), four patients (16.7%) had nausea and emesis, and three patients (12.5%) had weakness and fatigue. Hepatomegaly was present in eighteen patients (75%) and jaundice was present in only one patient (4.2%). Acute liver failure due to tumor infiltration in NHL has been reported earlier [11]. Higuchi et al. [12] reported the presence of chronic liver disease in 16 patients (31%) of the 51 cases of PHL.

It is rare for a patient with PHL to present with very high bilirubin levels. Jaundice in PHL patients can be due to the following reasons: (a) patients having a concomitant history of cirrhosis, (b) patients presenting with fulminant liver failure due to massive sinusoidal infiltration by lymphoma, and (c) lymphoma causing intra- or extrahepatic biliary obstruction. The patient that we described above had a progressive increase in total bilirubin reaching a peak value of 23.3 mg/dL. To the best of our knowledge, this is the first report described in literature where a patient with HCV infection had such a high bilirubin level due to PHL.

60% of the patients with PHL tested for HCV were positive [2]. Some of the possible theories for HCV induced lymphoma genesis include (a) continuous external stimulation of lymphocyte receptors by viral antigens and consecutive proliferation, (b) HCV replication in B cells with oncogenic effect mediated by intracellular viral proteins, (c) permanent B cell damage, for example, mutation of tumor suppressor genes, caused by a transiently intracellular virus (hit and run theory), and (d) prevention of B cell apoptosis by downregulation of caspase 1 and caspase 4 [13, 14].

Histological subtypes of PHL described in the literature include diffuse large B cell lymphoma (DLBCL), Burkitt's lymphoma, mucosa associated lymphoid tissue (MALT) lymphoma, mantle cell lymphoma, and T cell lymphoma with the most frequent one being DLBCL (96%) [10]. PHL has its own characteristic imaging features. Elsayes et al. [15] described 12 cases of PHL. Three patients (25%) presented with a single focal lesion, 8 patients (67%) presented with multiple well-defined lesions, and 1 patient (8%) presented with diffuse hepatic involvement on CT imaging. Prognosis of PHL patients with nodular infiltration of the liver is better than that of diffuse infiltrative pattern [6]. The patient that we described has nodular infiltration pattern of the liver involvement. Imaging studies in PHL can mimic that of metastatic disease, hepatocellular carcinoma, and liver abscess and hence liver biopsy is necessary to make the diagnosis. Tumor markers like AFP, carcinoembryonic antigen (CEA), and CA 19–9 are usually normal.

## 4. Management

The treatment of patients with PHL reported in the literature has not been consistent. Patients were treated with various modalities like surgery, combination chemotherapy, radiation therapy, or a combination of these. Localized PHL could be cured by resection, or the tumor burden could be reduced before or after surgery with chemotherapy [16]. Surgical resection was not feasible in our patient due to presence of multiple lesions.

A complete remission (CR) rate of 83.3% was achieved in PHL when systemic combination chemotherapy was used as the main modality of treatment [2]. Similarly, CR was achieved in all 4 cases of PHL in HCV patients after systemic chemotherapy. The presence of chronic active hepatitis on account of the HCV infection does not appear to influence response and tolerance to chemotherapy [17].

Adriamycin (one of the agents in CHOP regimen), an anthracycline, is predominantly excreted in bile. Patients with cholestasis have delayed clearance of doxorubicin and its metabolites and can lead to greater systemic toxicity [3, 18]. Despite reports that treatment with systemic chemotherapy can lead to complete response, it is not an option in our patient as his bilirubin is >20 mg/dl. Ma et al. [19] reported a patient with PHL whose total bilirubin was 20.87 mg/dL prior to initiation of an attenuated R-CHOP chemotherapy. Though the patient's total bilirubin improved to 1.19 mg/dL prior to the third cycle of chemotherapy, patient died from the complication of chemotherapy after fourth cycle. So patients with PHL and jaundice are a challenge in regard to the safest and best available modality of treatment.  Table 3 summarizes the cases of jaundice (bilirubin > 2 mg/dL) due to PHL reported in the literature and how they were managed on a Pubmed search. Cases where the management was not outlined were not included in the table. In our management, we have opted to give localized radiation therapy to reduce bilirubin levels, which thereby facilitated our use of R-CHOP chemotherapy. Our patient did have a complete response after six cycles of chemotherapy without any residual activity in the liver on the restaging PET scan. By administering chemotherapy, when the bilirubin was relatively mildly elevated, we think we might have prevented more serious complications from the chemotherapy. To the best of our knowledge, this is the first report in the literature where use of localized radiation therapy to the liver before systemic chemotherapy prompted a dramatic drop in bilirubin levels, thereby allowing the use of systemic chemotherapy (R-CHOP) and achieving a complete response in a PHL patient with HCV infection.

## 5. Conclusion

When multiple space occupying lesions are seen on imaging of the liver without any primary lesion or any other organ involvement with normal alpha fetoprotein, CA19–9, and carcinoembryonic antigen (CEA) levels, PHL should be considered in the differential diagnosis of these lesions and biopsy should be strongly considered in patients with HCV infection. PHL can cause significant hyperbilirubinemia in patients with HCV infection. If PHL is causing hyperbilirubinemia in a patient with HCV infection, localized radiation therapy can be considered to reduce bilirubin levels within an acceptable range where full dose of chemotherapy can be initiated in avoiding serious life-threatening complications.

## Figures and Tables

**Table 1:** Trend of patients LFTs.

Date (month/day/year)	Total bilirubin (mg/dL)	Direct bilirubin (mg/dL)	AST	ALT	ALP
11/21/2012	0.3		56	88	68
12/12/2012	6.1	4.1	150	142	282
12/22/2012	23.3	14.9	277	197	199

**Table 2:** LFTs trend table after initiating radiation therapy.

Date (month/day/year)	Total bilirubin (mg/dL)	Direct bilirubin (mg/dL)	AST	ALT	ALP
12/28/2012 **(day of radiation therapy)**	19.8	10.9	147	155	176
12/30/2012	10.3	5.4	103	127	140
01/10/2013	3.8	Not done	93	151	90
05/08/2013	1.3	Not done	71	69	85

**Table 3:** 

*n* = PHL + jaundice	Age, sex	Lesion	T bili mg/dL	Histology	Treatment	Outcome	Reference
1	67, M	Multiple nodules in liver	20.87	DLBCL	R-CHOP	Died after 4th cycle due to ARDS	Ma et al. [19]

1	62, M	Large solitary right hepatic lobe mass	N/M	DLBCL	None	Died 3 months after tumor diagnosis	DeMent et al. [4]

2	53, M	Diffuse	N/M	Pleiomorphic small cell*	Prednisolone and later multiagent chemotherapy	Died of disease at 18 months	Anthony et al. [5]
	76, M	Diffuse	N/M	T zone*	Prednisolone and chlorambucil	Alive and well at 3 years	

2	49, M	Nodular	N/M	B cell type	8 cycles of anthracycline containing CT^#^	CR at 52 months	Emile et al. [6]

1	90, F	Single mass in left lobe	2.3	DLBCL	4 cycles of attenuated CHOP	CR at 2 years	Agmon-Levin et al. [7]

1	55, F	Presented as biliary stricture with tumor in caudate lobe and left lobe of the liver invading CBD and occluding portal vein	3.4	DLBCL	Surgery followed by 6 cycles of CHOP + VP 16	Alive after 4.5 years after surgery	Yoneyama et al. [8]

N/M: not mentioned; M: male; F: female; CT: chemotherapy; DLBCL: diffuse large B cell lymphoma; *based on Kiel's classification subtype; ^#^complete chemotherapy regimen not mentioned; CR: complete remission; VP 16: etoposide, ARDS: acute respiratory distress syndrome, T bili: total bilirubin, mg/dL: milligram/deciliter, PHL: primary hepatic lymphoma; R-CHOP: rituximab, cyclophosphamide, adriamycin, vincristine, and prednisone; CBD: common bile duct.
